# Toward a Consensus Characterization Protocol for Organic Thermoelectrics

**DOI:** 10.1002/adma.202520430

**Published:** 2026-02-05

**Authors:** Bernhard Dörling, Ian E. Jacobs, Irene Brunetti, Nathan James Pataki, Shannon K. Yee, Dorothea Scheunemann, Keehoon Kang, Guangzheng Zuo, Juan Sebastián Reparaz, Takao Mori, Michael L. Chabinyc, Christian Müller, Mario Caironi, Martijn Kemerink, Mariano Campoy‐Quiles

**Affiliations:** ^1^ Institut de Ciència de Materials de Barcelona ICMAB‐CSIC Bellaterra Spain; ^2^ Optoelectronics Group Cavendish Laboratory University of Cambridge Cambridge UK; ^3^ Institute for Molecular Systems Engineering and Advanced Materials Heidelberg University Heidelberg Germany; ^4^ InnovationLab GmbH Heidelberg Germany; ^5^ Center for Nano Science and Technology Istituto Italiano di Tecnologia Milano Italy; ^6^ George W. Woodruff School of Mechanical Engineering Georgia Institute of Technology Atlanta Georgia USA; ^7^ Materials Science and Engineering Seoul National University Seoul Republic of Korea; ^8^ Institute for Electric Light Sources College of Intelligent Robotics and Advanced Manufacturing Fudan University Shanghai P. R. China; ^9^ Research Center For Materials Nanoarchitectonics National Institute for Materials Science Tsukuba Japan; ^10^ Materials Department University of California Santa Barbara California USA; ^11^ Department of Chemistry and Chemical Engineering Chalmers University of Technology Göteborg Sweden

**Keywords:** best practices, organic thermoelectrics, Seebeck coefficient, thermal conductivity, thermoelectric characterization, thermoelectric generators

## Abstract

As the field of organic thermoelectrics advances toward maturity, an accurate and standardized reporting of performance metrics becomes essential to drive further progress and assess real‐world viability. The common geometric form factors and material properties (conductivity, anisotropy, stability, etc.) differ from those of conventional bulk inorganic systems, and thus specific recommendations may apply. Herein, we compile prevalent points of concern in the reporting of thermoelectric performance for organic materials and devices. Moreover, we propose a list of critical factors and metrics that should be explicitly documented when reporting the performance of novel organic thermoelectric materials or devices.

## Graphical abstract

We advocate a common consensus on accurate and standardized reporting of performance metrics in the field of organic thermoelectrics. We summarize prevalent issues in the literature and propose a pre‐submission checklist to support the publication of reproducible results.

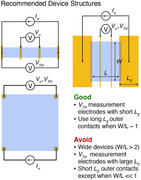

## 1 Introduction

The solid‐state conversion of heat into electricity through the thermoelectric (TE) or Seebeck effect has been known for over 200 years. It was, however, during the 1950s when practical applications started to emerge, and figures of merit (FoMs) to characterize thermoelectric materials and generators were established [1]. The thermoelectric material figure of merit *zT = S^2^σT/κ* at a given temperature (*T*) combines other properties that are relevant to assess performance, namely the Seebeck coefficient (*S*), the electrical conductivity (*σ*), and the thermal conductivity (*κ*). While these all have a very clear physical definition, their characterization may be challenging in real samples and operational conditions.

Inorganic thermoelectric materials are mainly characterized in their bulk form (as single crystals, ingots, or pellets with similar length, width, and thickness), and often far above room temperature. The associated complications in reporting meaningful FoMs are mostly well identified and accounted for in bulk inorganic samples. Very recently, an International Standardization Organization (ISO) norm has been established to simultaneously measure *σ* and *S* from 300 to 1200 K in bulk samples [2]. Similarly, there are ISO norms governing the determination of the thermal conductivity of bulk samples [3].

The field of organic thermoelectrics presents its own unique set of challenges relative to inorganic materials. Organic materials are frequently cast from relatively dilute solutions or deposited from vapor into thin films (with a thickness of typically less than 1 µm). As synthesized, they may be highly insulating, requiring the addition of chemical dopants via further processing steps. Their electrical and thermal conductivities can span orders of magnitude, and, depending on processing, may be anisotropic. Last but not least, organic materials tend to have significant air stability issues. These traits necessitate the development of and adherence to a tailored set of guidelines, to assure that the reported FoMs for materials and devices are meaningful, comparable, and valuable to the scientific community.

In the ideal, hypothetical case, any thermoelectric material should be characterized by measuring *S*, *σ*, and *κ* at the same time, in the same direction, and on the same homogeneous sample.

Given experimental and other limitations, in common practice however, multiple, and not necessarily perfectly homogeneous samples are measured sequentially. While it is often implicitly assumed that these distinct samples possess identical and unchanging properties, this is not necessarily the case. Additionally, in anisotropic materials, an erroneous *zT* may result for thin‐film samples, when *σ* is measured in the in‐plane direction, while *κ* is measured in the out‐of‐plane direction. This issue may be mitigated by measuring *κ* on the same (or an identical) thin film sample in the same in‐plane direction as *σ* [4]. Care should be taken if multiple samples are used, because the degree of anisotropy depends on the sample morphology, which in turn can be influenced by film thicknesses, as often seen through optical studies [5], as well as by thermal [6], and structural characterization [7].

Likewise, when comparing the performance of individual samples to that of organic thermoelectric generators comprising multiple elements, numerous parameters (e.g. processing conditions, measurement apparatus, sample geometry, measurement direction, contacts, fill factor, etc.) change at once, which can lead to inconsistent results if not properly accounted for and documented.

The scenario outlined for organic thermoelectrics resembles challenges that are common for any emerging material system, due to their often multidisciplinary nature. Recent examples include the domains of organic photovoltaics (OPVs), organic field effect transistors (OFETs), hybrid perovskite photovoltaics, and photodetectors based on emerging semiconductors. The early days of OPVs saw claims of overly optimistic performance, some of which were due to experimental errors, such as issues related to solar simulator calibration, or an ill‐defined device active area [8]. The prevalence of these systematic errors in the literature was quantitatively discussed by documenting the mismatch between reported short circuit currents and the corresponding external quantum efficiencies integrated over wavelength [9]. In the realm of hybrid perovskite PVs, challenges were compounded by hysteresis in the current‐voltage characteristics, that could, depending on the direction and scan rate of the voltage sweep, result in misleadingly large fill factors and inflated power conversion efficiencies [10]. Consequently, the PV community implemented standardized reporting protocols, as evidenced by guidelines instituted by journals such as Nature [11], Energy & Environmental Science [12], or Advanced Materials [13]. Additionally, a consensus was established regarding the reporting of device stability for both OPV [14], and perovskite PV [15], facilitating more straightforward comparisons across different studies. Similarly, as research into OFETs gave rise to the development of enhanced materials, measurement methods based on simplistic models had to be expanded to take into account sample‐dependent effects when extracting carrier mobility [16, 17]. Also, a broad community of researchers engaged with the development of photodetectors based on novel materials, called for [18], and reached a consensus on proper optoelectronic characterization, which requires specialized approaches to obtain reliable and meaningful FoMs [19].

Given that the field of organic thermoelectrics is reaching maturity, and best‐reported materials are becoming competitive with inorganics [20], we think it is both timely and essential to standardize protocols for reporting relevant performance and stability parameters.

In this perspective, we aim to compile prevalent issues in the reporting of FoMs for organic materials and their associated devices alongside potential solutions into a concise report. While some of these issues and solutions have been well‐established and may be common knowledge, others have emerged more recently. With the aim of fostering rigorous and complete reporting of new findings in the field, we decided to provide a systematic overview of critical factors and metrics that we recommend should be explicitly documented when reporting novel organic thermoelectric materials or devices. We consolidated these into a preliminary checklist (provided as Supporting Information), intended to serve as a foundation for further discussion. We hope this ultimately yields a coherent set of principles for the consistent characterization and reporting of organic TE materials and devices.

## 2 State of the Art in Bulk TE Samples Characterized at High Temperature

The field of inorganic thermoelectrics predates that of its organic counterparts, and a considerable body of work exists that details commonly agreed‐upon best practices when working with bulk materials [21, 22], or complete devices [23]. Even so, recent assessments suggest typical measurement uncertainties for *S*, *σ*, *κ*, and *zT* may be as large as 10%–20% [24], serving as a reminder that even in a mature field, errors can be significant and should always be reported together with corresponding information on instrumentation and protocols. To provide context for sources of the error, reported uncertainties should be accompanied by the respective sample population, that is, the number of devices, samples, or batches that have been prepared and analyzed. It should be stated if any outliers were excluded from the statistical analysis, and the omitted data should be provided separately.

Specific challenges related to the relatively high temperatures that are typical for inorganic TE materials are mostly well‐known and documented extensively elsewhere [25]. Temperature measurement inaccuracies, in particular, cannot be neglected for some methods [26]. Similarly, errors stemming from asynchronous temporal and spatial measurement of voltage and temperature or the effect of contact geometry and quality are well documented, and can be estimated [27]. When analyzing the acquired raw data of Seebeck measurements, some best practices and consistency checks may help increase their accuracy [28]. While some of these documented best practices are strictly necessary only when measuring at high temperatures, most of them can be useful to improve measurements of bulk samples at any temperature.

Finally, given that inorganic thermoelectric materials have a long history, the National Institute of Standards and Technology (NIST) provides Standard Reference Materials for the Seebeck coefficient: bulk samples for low temperatures from 10–390 K (SRM 3451) and for high temperatures from 295–900 K (SRM 3452). Going beyond homogeneous samples, more complex characterization techniques that can measure *S* and *σ* locally have been demonstrated, and proven useful in the high‐throughput characterization of combinatorial films [29]. Importantly, the availability of proper reference samples facilitated the validation of the aforementioned techniques. Conversely, the substantial sample‐to‐sample variability and limited stability of typical doped organic semiconductor samples seem, at present, to preclude establishing a similar organic‐based reference for organic thermoelectrics. Instead, the field typically relies on a variety of easily available inorganic references that are more or less suitable for the range of interest (e.g. nickel or constantan for *S*, or glass for out‐of‐plane *κ*). However, whenever these references differ significantly from the actual samples in terms of geometric, thermoelectric, or mechanical properties, there is more room for error.

In our view, this highlights the critical importance to comprehensively document experimental and other procedural details, such that research can be more easily evaluated and reproduced.

## 3 Improving the State of Affairs in the Characterization of Organic Thermoelectrics

Besides the already mentioned points, the characterization of the thermoelectric properties of organic materials has to address additional idiosyncrasies. In the following, we illustrate this by presenting cases of challenges and their proposed solutions as reported in the literature. We want to stress that our purpose with these examples is to represent general classes of issues pervasive across the literature rather than to criticize specific instances. By no means is it our intention to cast doubt on the overall validity of the main conclusions in the cited works. Our approach has been to proceed from the general to the specific; in this case from the raw material to the prepared samples, then to the different measurement methods, and concluding with some final considerations on self‐consistency.

### 3.1 Description of Materials

The most thorough characterization is incomplete, if the material under study is not sufficiently well‐defined and documented. The supplier, and if possible, the batch number of the material should be provided. Alternatively, if published, a literature reference to the synthesis procedure should be provided. For polymers, the molecular weight and dispersity should be specified because these properties are known to significantly influence *σ* and *S* [30], as well as *κ* [31].

#### 3.1.1 Sample Preparation and Geometry

Many research labs that study organic thermoelectric materials typically focus on thin‐film samples, relying on their expertise in a wide variety of solution deposition techniques (e.g. spin‐, blade‐, and spray‐coating, inkjet printing, etc.) [32]. In the field of organic electronics, these techniques are widely used to deposit films with a thickness of the order of 100 nm, and myriad characterization techniques are available for studying their morphology and optical properties. Less common, though no less important, bulk samples such as micro‐ to millimeter‐thick films, fibers, yarns and foams are widely studied within the context of organic electronics since many classical polymer processing techniques (e.g. melt pressing, fiber spinning, etc.) readily provide access to these geometries. Last but not least, samples made from aerogels [33], cryogels, or hydrogels [34, 35, 36], have recently become of interest due to their peculiar properties. To help interpret results, it is therefore important that sample preparation and doping methods should be described in detail, including any additional post‐processing steps. For instance, different techniques such as solution‐ and vapor‐doping of thin films yielded substantially different morphologies and TE performance, even when using the same polymer and dopant [37, 38, 39]. In case of bulk samples, doping via a post‐processing step can lead to doping gradients because of limited mass transport of the dopant through thick layers [40]. Moreover, care should be taken when comparing the thermoelectric properties of samples prepared with different methods, e.g. thin films vs. fibers, since those can feature very different nano‐ and microstructures. Providing only limited information on sample preparation complicates the interpretation of seemingly conflicting reports, as for example in the case of dimethyl sulfoxide (DMSO)‐treated poly(3,4‐ethylenedioxythiophene):poly(styrene sulfonate) (PEDOT:PSS), where significant variations of *κ* were observed for ostensibly equal *σ* [41, 42, 43]. Even seemingly commonplace procedures, such as using polypropylene pipette tips, or working in a glovebox that is shared across experiments, can lead to unintentional contaminations that have been shown to have a noticeable effect [44].

Something as basic as the size of a sample can be important, particularly in relation to the size, location, and shape of the contacts. As will be established in the section on measurement methodology, sample dimensions and contact layout should always be specified. Similarly, with regard to thin films, a large surface roughness, non‐uniform film thicknesses, non‐uniform doping density across lateral and vertical directions of the doped films, film porosity, or incomplete coverage can all directly or indirectly influence the measurement results. Furthermore, when using optical characterization techniques, the above non‐idealities can affect light scattering which complicates absorbance measurements. The following discussion will primarily focus on thin‐film samples, since those represent the most widely studied sample geometry. Even so, we will provide references for bulk samples in specific cases, where the extensive literature on bulk inorganic thermoelectrics summarized in Section 2 is not directly applicable.

#### 3.1.2 Environmental Conditions

The environmental conditions to which samples are exposed both during, as well as prior to measurements, should be documented, especially the atmospheric conditions and temperature. Humidity, in particular, is known to affect the electrical properties of films through mechanisms such as the introduction of oxygen‐ or water‐induced trap states [45, 46], or ionic conduction [47]. In the past, the effects of uncontrolled humidity levels were a factor in some reports of record *zT* values [43, 48]. Despite a follow‐up report that investigated the impact of humidity under controlled conditions, the initial findings could not be satisfactorily reproduced [49]. This hints at additional challenges, such as significant lab‐to‐lab and batch‐to‐batch variability of materials. Lastly, measurements should best be conducted in the dark, to avoid any potential contribution due to photogenerated charges [50, 51, 52, 53].

#### 3.1.3 Stability

When investigating stability over time via repeated measurements, detailed knowledge of the environmental conditions becomes even more important. Only if the complete protocol that samples pass through during and between measurements is known can useful information about stability be inferred. In addition to these external factors, sample properties may also play a major role. For example, the apparent stability of doping can strongly depend on film thickness. For carbon nanotube films, it was explicitly shown that by varying film thickness, the characteristic timescale of degradation for a given dopant can be changed by several orders of magnitude [54], a behavior which can reasonably be expected to also occur in organic films. This overlooked fact alone is sufficient to explain why for any given carbon nanotube dopant there are reports that find both stable [55], and unstable behavior [56], mistaking an incidental sample property for a fundamental material property.

### 3.2 Measurement Methodology

Having covered the “why,” “what,” and “when” of thermoelectric characterization, we now turn to the “how.” While there is literature on thin film characterization methods of TE samples [57, 58], many research groups engaged in the study of organic thermoelectrics build and use their own, often scantily documented setups. While some of these instruments are subsequently documented in the literature [59, 60, 61, 62] or made available commercially [62], many are only ever detailed by sketches provided as Supporting Information, making it difficult to further build upon the work. Hence, each employed measurement method should be sufficiently described to allow for reliable reproduction of experiments. For widely recognized methods and commercial instruments, this may be as simple as giving their full name or model. In contrast, for non‐standard or newly developed methods, and custom‐made/modified instruments, a literature reference or a comprehensive description should be included. In particular, it should be expressly stated which reference samples were used, or how the measurements were validated.

#### 3.2.1 Thermoelectric Measurements

When measuring electrical conductivity, one should be wary of excessive contact resistances, though they can easily be avoided by using two contacts in combination with the transfer length method [63], or ideally, a four‐point probe method [64]. While the latter is preferred for films, fibers have been characterized using both methods [65]. The contact resistance as well as the apparent Seebeck coefficient can be strongly influenced by the choice of electrode material [66], which was attributed to interfacial doping. A particularly simple method to determine *σ* is the van der Pauw method, as well as its extension to anisotropic materials, the Montgomery method [61, 67, 68], which are typically used with homogeneous, rectangular, hole‐free films with contacts placed at the corners. While other methods may seem to allow for more freedom when placing electrodes, care should always be taken when contacts are situated within a continuous film, as opposed to at its edges. Methods based on the former typically require geometry‐dependent correction factors, which should always be documented [64]. Failing to account for such edge effects may induce systematic errors, for example, in the form of overestimating the degree of anisotropy of *σ* [68], or the magnitude of *S* [48].

**FIGURE 1:**
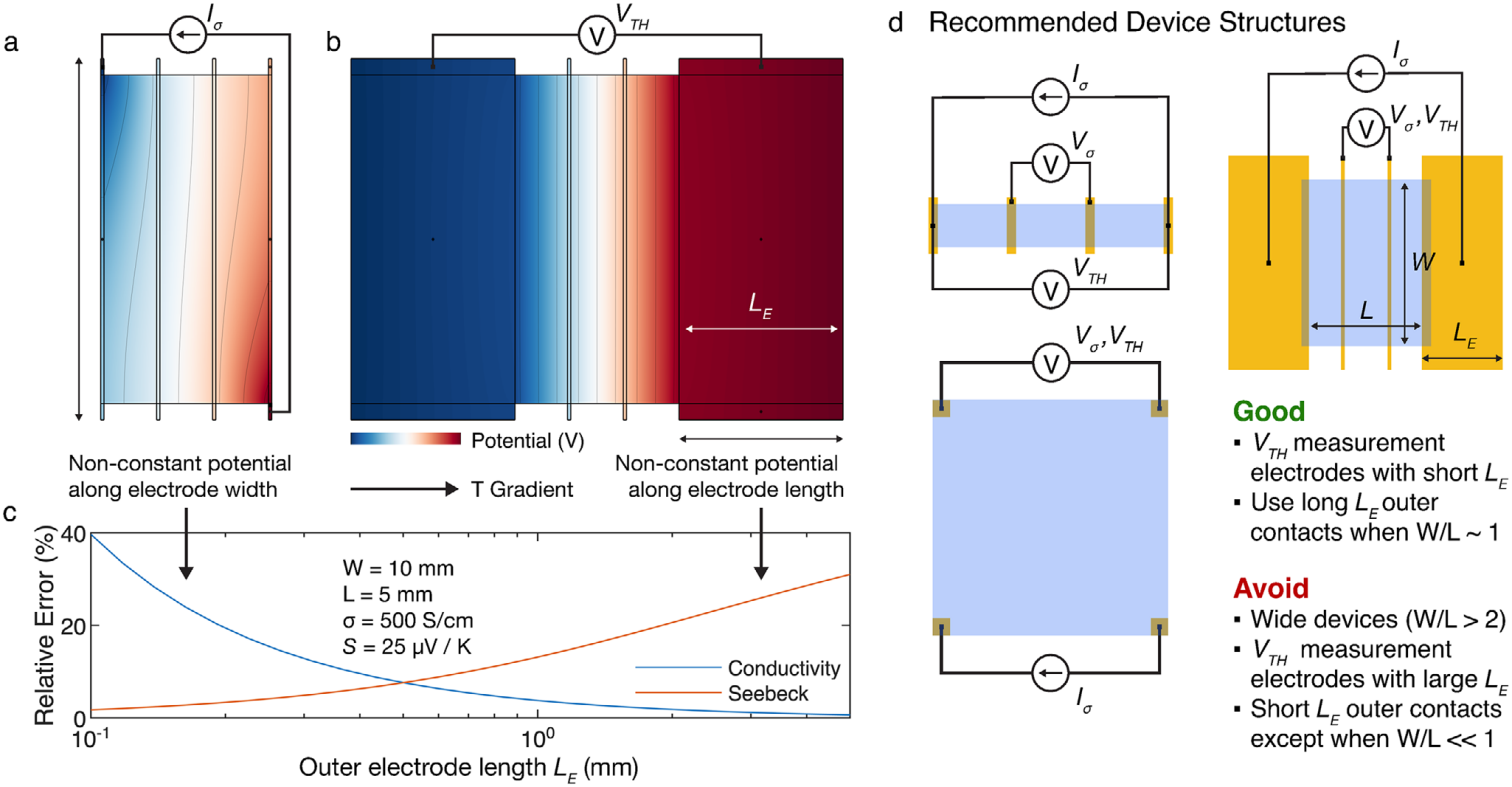
Systematic errors can occur in both electrical conductivity and Seebeck coefficient measurements as a result of the finite conductivity of electrodes. (a) Finite‐element method calculation of the potential in a device composed of a polymer thin film (50 nm thick, *σ* = 500 S/cm, *S* = 25 µV/K) with gold electrodes (25 nm thick, *σ* = 1.6 × 10^5^ S/cm, *S* = 1 µV/K). To illustrate the problem, current source contacts are made at opposite corners of the device. The resulting non‐uniform potential gives rise to a large error in conductivity. (b) Potential map of a thermovoltage measurement in a similar device with longer outer electrodes. A potential gradient along the length of the electrode appears due to the thermovoltage of the electrodes, leading to an error in the measured Seebeck coefficient. (c) Comparison of the relative error in *σ* and *S* as the outer electrode length *L_E_* is varied. Temperature measurements are taken at the same location as the thermovoltage. (d) Recommended device geometries that minimize common errors. Films should be patterned such that any active material is confined between the electrodes. If *W*/*L*∼1, the outer, current‐carrying electrodes should be long. Contacts used to measure the thermovoltage *V*_TH_ should always be small (*L*_E_<<*L*). Contacts in van der Pauw geometry should be small and located in the corners.

Yet even in well‐patterned, four‐contact devices without any superfluous material, improper electrode geometry can produce large errors in *S* and *σ* [69], as shown in Figure 1. Particular care is required when characterizing high‐conductivity materials. In wide devices (with an aspect ratio *W*/*L* > 1), the electrode resistance along the width (*W*) of the device can begin to approach the resistance across the length of the channel (*L*). In this case, the potential in the device is no longer uniform but instead can depend on the position of the electrical probes and the geometry of the electrodes (Figure 1a). In a four‐probe configuration, this non‐uniform potential can lead to unbounded over‐ or underestimates of the true film conductivity, depending on the placement of the probes (Figure 1c). Using longer, more square‐shaped outer electrodes in four‐probe measurements reduces this error, but devices with aspect ratios *W*/*L* > 1 are not recommended for high conductivity materials. These considerations are particularly important when evaluating anisotropic samples, as some approaches are intrinsically better suited than others [68], with documented cases of significant overestimation of *σ*, due to suboptimal line‐shaped electrodes [68, 69].

Device geometry is important to Seebeck measurements as well. If the device is placed in a temperature gradient, and the electrodes, at which the thermovoltage is measured, are long relative to the device channel length, then the thermovoltage measurement will depend on the exact location of the probe contact points, since an additional contribution can develop within the electrodes (Figure 1b). In addition to this error in thermovoltage, if the temperature is measured at the probe contact points, as typically recommended, long electrodes can also produce an overestimation of the temperature difference along the channel. These combined effects can produce a significant error when measuring the Seebeck coefficient (Figure 1c). Figure 1d depicts some recommended device structures that allow to minimize these types of errors.

#### 3.2.2 Seebeck Coefficient Measurements

For organic TE materials, which are constrained to a narrower temperature range compared to their inorganic counterparts, the Seebeck coefficient is typically only measured at room temperature, rather than across an extended range of temperatures *S(T)*. To ensure an accurate interpretation and comparison of *S*, a pair of temperatures at which the measurement was conducted should be reported, such as *T*_avg_, and *ΔT*; or *T*_hot_ and *T*_cold_.

##### 3.2.2.1 Temperature Measurements

For accurate results, the temperature difference *ΔT* should ideally be determined in such a way that it matches the corresponding thermovoltage measurement [26, 27]. For short contacts with *L*_E_<<*L*, this effectively means that temperature and voltage should both be measured at the same location—on the contact. If *L*_E_ is larger, then the temperature should be measured at the inner edges of the measurement electrodes, because that is where the thermovoltage of the sample starts being generated. If this is not feasible, then spatial interpolation of the temperature profile may be employed. However, assuming a simple linear temperature gradient between contacts of homogeneously constant temperature may not be optimal, and a nonlinear temperature gradient could provide a more accurate description [70]. Likewise, if *V*(*t*_1_) and *T*(*t*_2_) cannot be acquired simultaneously (*t*_2_ = *t*_1_ + *Δt*), multiple measurements should be interpolated in time, to approximate concurrency and minimize errors due to drift (for example in the case of temperature, *T*(*t*_1_) ≈ (*T*(*t*_1_−*Δt*) + *T*(*t*_1_ + *Δt*))/2 [26]. Another way to acquire *V* and *T* simultaneously is to use a second set of leads (with its corresponding read‐out electronics). However, since the additional leads affect the temperature distribution in the sample [26], and because they cannot be placed at exactly the same location as the first set, this approach effectively trades reduced errors due to temporal drift for increased error due to spatial inhomogeneity, a phenomenon known from high‐temperature measurements, which is sometimes termed the cold finger effect. In any case, it should always be documented where and with which method the temperature was measured, and which assumptions were made. To avoid unnecessary issues, it should be explicitly checked that both probes agree over the temperature range of interest (*T*_cold_ < *T* < *T*_hot_), when no gradient is applied. This is of particular importance when temperatures are measured using IR thermometry. In that case, it should be reported how emissivity, which is dependent on the electrical conductivity, and thus on both the material and the doping level, was determined, since it may not be constant over the sample, or over time. Instead of measuring temperature directly, it is also possible to measure the thermovoltage generated by a known internal reference that is placed alongside the sample, thermally in parallel. This method is sometimes used when the reference and sample geometry can be matched, as in the case of a constantan wire reference and a thermoelectric yarn sample [63, 65].

##### 3.2.2.2 Thermovoltage Measurements

**FIGURE 2:**
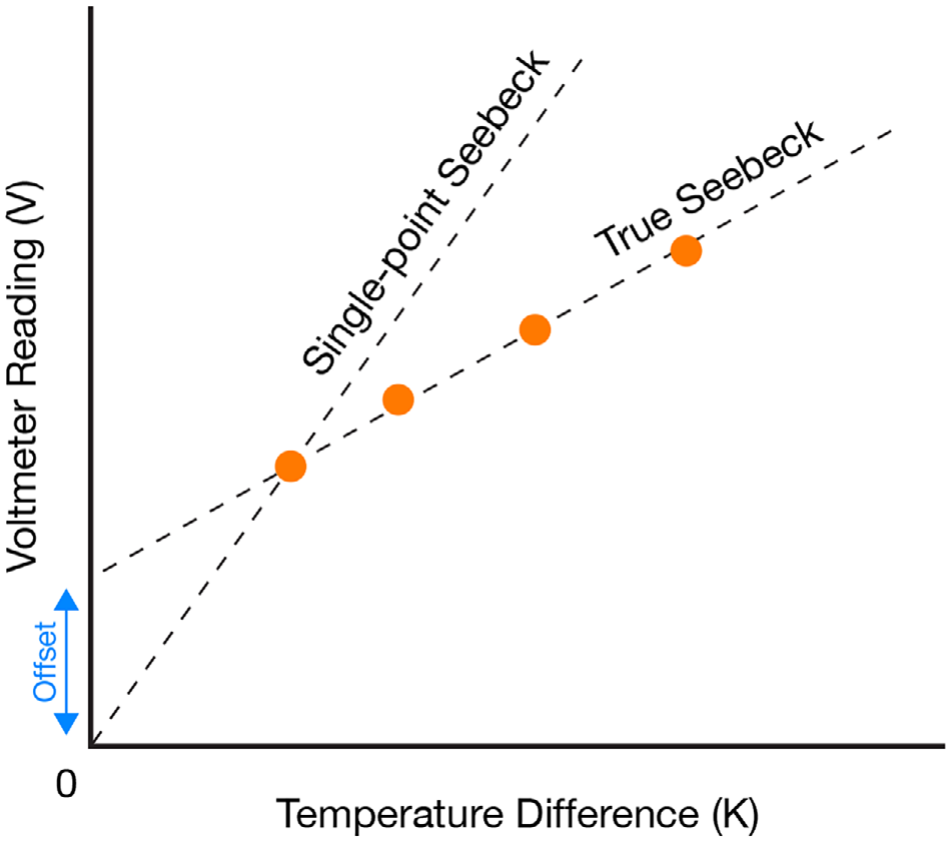
Stray voltages can cause a voltmeter offset. To accurately determine the Seebeck coefficient *S = V/ ΔT*, it should be obtained as the slope fitted to multiple *V‐ΔT* pairs. Assuming that *V* = 0 at *ΔT* = 0, and obtaining *S* from just a single measurement will typically give a wrong result.

When measuring small voltages, temperature‐dependent contact voltages between various components of the measurement circuit and/or the sample can be a challenge, particularly when the measurement is conducted at small temperature differences [71, 72]. *S* should never be determined by just measuring the bare minimum number of *V‐ΔT* pairs, and it should not be assumed that *V* = 0 at *ΔT* = 0, since stray voltages *V*_bias_ (e.g. thermovoltages generated due to the voltmeter warming up during operation) may induce an offset as sketched in Figure 2. Instead, accuracy should be increased by measuring many *V‐ΔT* pairs [71]. Alternatively, pairs of measurements ((*V* ^+^_hot_, *V* ^−^_cold_), (*V* ^−^_hot_, *V* ^+^_cold_)) can be used to remove the effect of *V*_bias_, either by inverting the temperature gradient *ΔT →—ΔT* to ascertain *V* from the combined temperature interval [72], or less commonly, by inverting the polarity of the electrical connection [61]. Additionally, another highly effective, albeit unsophisticated way to further reduce errors due to stray voltages is to simply increase *ΔT* and thereby the corresponding *V* signal [27]. While this may come at the cost of a less accurate determination of *S(T)* as a function of temperature, it more closely matches the conditions that a generator would encounter.

Measurements of highly resistive samples suffer from increased noise, which may render conventional methods impractical above sample resistances of the order of several MΩ. Using cyclic temperature gradients enabled measurements of samples with resistances up to around 100 GΩ [73, 74]. In any case, for a valid measurement, the sample resistance should always be small relative to the input impedance of the voltmeter used to measure the thermovoltage, necessitating specialized equipment such as electrometers.

##### 3.2.2.3 Ionic Effects

When characterizing materials with potentially mobile ions such as hygroscopic polymer films [47, 75, 76], and hydrogels [34, 35, 36], care should be taken to properly account for any additional frequency‐dependent response resulting from the thermodiffusion of mobile ions due to the Soret effect [47, 75, 76], or the thermogalvanic effect, which involves additional redox reactions at the electrodes, and that is beyond the scope of this work [34, 35, 36]. Such contributions, which can be strongly temperature‐dependent, may for instance lead to the overestimation of *S* or *σ*. Depending on the involved frequencies and timescales in AC and DC measurements, respectively, a large thermovoltage or current may mistakenly be attributed to the displacement of free charges instead of sample‐bound ions. When ionic transport is not the principal focus of the reported research, ionic contributions should be avoided by choosing appropriate experimental conditions. In simple terms, when working with ionic materials, gradients (of electric potential when measuring *σ*, or of temperature when measuring *S*) should be maintained for a long time (potentially up to hours). Comparing measurements that are taken over time then allows to determine how big the contribution of transient effects is, if any. To leave no room for doubt, characteristic timescales of the measurements, such as typical durations, ramp, and hold times should be specified along with explicit data or statements quantifying temporal stability and, where appropriate, correction schemes to disentangle the various contributions.

#### 3.2.3 Thermal Conductivity Measurements

The measurement of thermal conductivity presents comparatively difficult challenges, which has led to the development of numerous methodologies [77]. Not all of these measure *κ* directly. For example, some first measure the thermal diffusivity *α* and require further inputs, such as density and specific heat capacity. In these cases, it should be explicitly stated how every parameter was independently measured, or if any were taken from the literature.

For highly electrically conducting materials, the electronic contribution to the thermal conductivity, *κ*_el_, and the electrical conductivity are often not independent, but instead typically follow a linear relation called the Wiedemann–Franz law *κ*_el_ / *σ* = *LT*, with a proportionality constant called the Lorenz number *L*, which typically takes a value around the Sommerfeld value L_0_ = *π* ^2^/3 (*k*_b_/*e*)^2^. Using DMSO‐treated PEDOT:PSS as an illustrative example, one can find reports that agree with *L*_0_ [41], while others report larger values *L* > *L*_0_ [42], or data consistent with smaller values *L* < *L*_0_ [43]. While neither observation can upfront be discarded on the basis of theoretical considerations [78], it can be unclear if observed differences are due to differences in sample preparation, environmental factors, or simply inaccurate characterization. Alternatively, it is plausible that the differences can be attributed to inhomogeneity that complicates measurements, analyses, and interpretations [79], or to doping‐induced changes in the microstructure [80].

**FIGURE 3:**
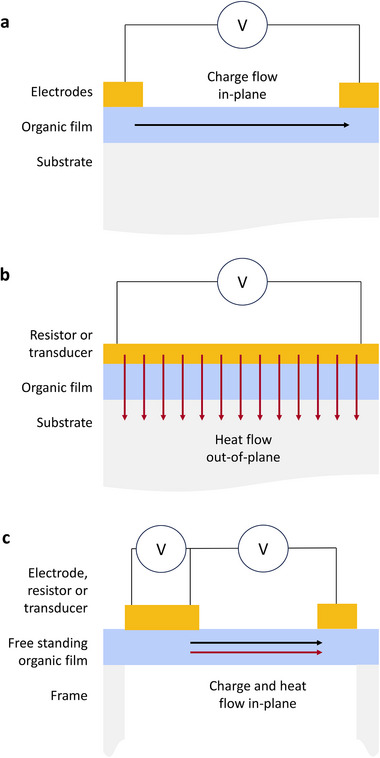
(a) In a typical electrical conductivity measurement, charges flow between the electrodes along the plane of the substrate, and thus the *σ* component accessed is in‐plane. (b) In standard thermal conductivity measurement techniques, such as 3‐*ω*, thermoreflectance, and Raman thermometry, heat is generated electrically in a resistor, or optically in a transducer (not pictured), and flows toward the substrate, thus the out‐of‐plane component of *κ* is accessed. (c) Special samples (e.g. based on thin supporting membranes or free‐standing films) or advanced measurement techniques can be used to enhance the heat flow in the in‐plane direction.

In thin films supported on substrates, heat locally flows mainly from the heating element (resistor heated by electric current, transducer heated by light beam, etc.) toward the substrate, as sketched in Figure 3. This implies that, unless specific geometries or samples are used, the effective thermal conductivity that is accessed is approximately the out‐of‐plane component of the thermal conductivity tensor. When samples are isotropic and films are sufficiently thick (compared to the phonon mean free path), this is not an issue. When samples exhibit some degree of anisotropy (either microstructural or due to geometrical confinement), this could be an important source of error when correlating *κ* to *S* or *σ*, since those are almost always measured in‐plane. As most solution‐processed polymers tend to exhibit a preferential orientation of the backbones in the plane of the substrate [81, 82], their in‐plane conductivities are expected to be higher than those of out‐of‐plane [31]. Therefore, measuring *σ* in‐plane and *κ* out‐of‐plane will systematically overestimate *zT*. Instead, *κ* should be measured in‐plane, for example, on freestanding samples using a suitable method such as thermoreflectance [83], particularly with a 1D heat source [84], as well as variations of the 3‐ω method [4]. When working with bulk samples with unconventional geometries or architectures on the other hand, suitable approaches include thermoreflectance adapted to fibers [85], or the transient plane source method, which can be used with bulk samples, including porous structures such as foams [86, 87], provided the thermal contact resistance is properly accounted for [88].

#### 3.2.4 Thermoelectric Generators

Besides investigating materials, many reports include data on a thermoelectric generator (TEG) comprising multiple p‐ and n‐type legs, which typically necessitates separate equipment to characterize. Enough information should be provided to put its performance in context and facilitate the reproduction of results. Merely reporting absolute output power is insufficient, and a few key factors have to be reported. The number of TE pairs that constitute the generator provides an indication of the scalability of the approach. Devices composed of only a few TE legs, while potentially showing high performance, may reflect underlying limitations in the scalability of the fabrication process or materials integration. The total dimensions of the generator are needed to calculate power density (power/area), TEG power factor (power/area/temperature^2^), and other normalized indicators that enable comparison between results [89, 90, 91, 92]. In particular, it should be emphasized that densities should be calculated using the device footprint, that is, the total area they occupy on the heat exchangers. They should not be normalized by the fill factor or calculated from the active areas only.

The output power should be determined by measuring the thermovoltage vs. a variable load resistance [90, 91], or by varying the bias current [63, 89], to confirm the expected parabolic behavior. In particular, measurements should always include those across a load resistance matched to the internal resistance, and across an open circuit, corresponding to the maximum power point and the Seebeck voltage respectively. Additionally, power should be measured for varying temperature differences [90, 92], to again confirm the expected quadratic dependence on temperature. If mobile ions are an issue, then a measurement over a sufficiently long timescale, that reaches a stable plateau, which either demonstrates or disproves ionic conduction, should be included [76, 93]. So‐called ionic thermoelectrics, where ionic contributions are significant, should be treated separately. They typically exhibit a superficially large but transient power factor, as sample‐bound ions start to accumulate at one electrode. Since ionic thermoelectrics as well as thermogalvanic cells then require some form of regenerative procedure to continue performing (e.g. an inversion of the temperature gradient) [76], they should be considered their own categories, separate from regular, maintenance‐free TEGs.

It should be discussed whether the TEG performance is consistent with the thermoelectric properties of the constituent active materials. Typically, observed deviations are due to three reasons. First, the generator active material may not reach the performance that was reported for single‐material samples, which is often due to reproducibility issues when scaling up the amount of material. Second, electrical contact resistances in the generator are often larger than expected [94]. And third, the temperature difference applied to the generator may be very different, often much larger, than the one used for single‐material samples, or it may be inaccurately determined. Ideally, the fraction of the temperature difference that drops across the active materials (as opposed to the complete stack) should be measured or estimated [33, 90, 91, 95]. Key factors when modeling *ΔT* include the thickness and thermal conductivity of the leg, the metal contacts, and the substrate, as well as the fill factor.

When evaluating the long‐term performance and stability of the device, assessments should involve repeated characterization over a specified time, explicitly reporting the relative change compared to the initial state [89]. Similarly, when evaluating reproducibility, the number of devices should be specified, and the relative spread of performance should be reported [96]. It is imperative to comprehensively specify the environmental conditions during these tests, including atmosphere (most importantly relative humidity), and the temperature [89]. Details on additional factors, such as encapsulation methods, exposure to mechanical stress, or other relevant stressors, should also be included as necessary to ensure a comprehensive understanding of the device stability.

A supplementary piece of information that is useful to gauge the design of the generator is how the temperature gradient is established and maintained during the characterization. Particularly for large applied *ΔT*, it is instructive to know if active cooling or an external heat exchanger were employed to sustain the reported temperature gradient, or if natural convection was sufficient. We encourage authors to provide photographs of devices taken during measurement.

### 3.3 Consistency Checks

**FIGURE 4:**
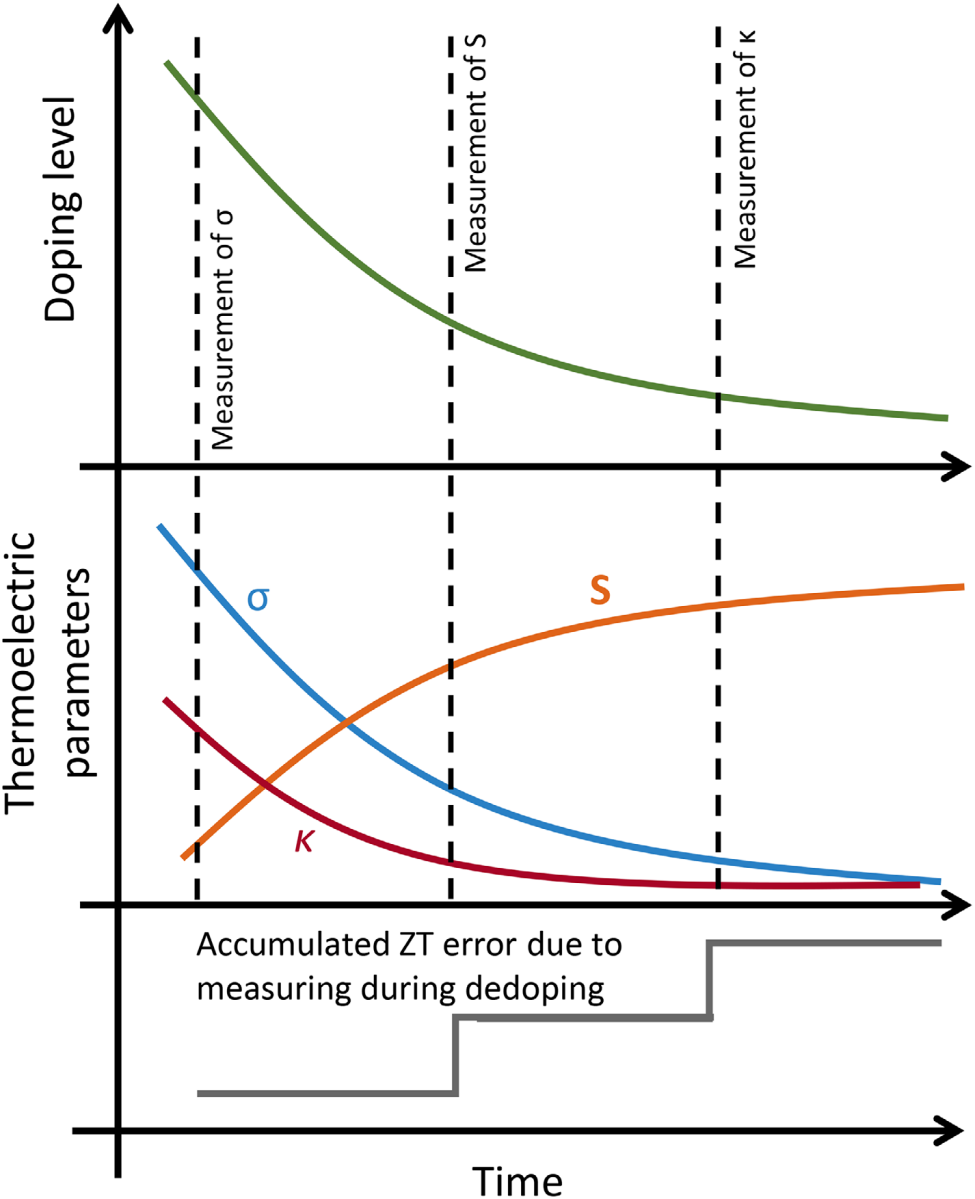
Sketch of how the thermoelectric parameters change over time in a sample that is spontaneously dedoping. A typical measurement sequence, in which *σ* is measured first, *S* second, and *κ* at the end, will result in an overestimation of *zT*.

Having all this data at one's disposal, both the diligent author as well as the interested reader can more readily verify if results are self‐consistent. A critical aspect to confirm is that all the samples subjected to the various characterization techniques can reasonably be assumed to have similar, if not identical, properties. For instance, one cannot necessarily assume that a bulk sample, as is sometimes used to measure *κ*, possesses the same properties as a thin film sample used for the measurement of *S* or *σ*. The degree of anisotropy, as well as the doping level may strongly vary. That is why for each evaluated parameter, the corresponding measurement direction should always be explicitly specified. Likewise, one cannot presume upfront that a material that was characterized for its thermoelectric properties in isolated thin films will exhibit the same characteristics when integrated in a demonstrator TEG device. As such, the information provided for both materials and demonstrator should enable the reader to estimate any deviations, ideally with such deviations explicitly discussed in the manuscript. Measuring particularly unstable samples (e.g. doped with reactive, or volatile compounds) may result in misleading values for *S* ^2^ *σ* or *zT*. Since *S*, *σ*, and *κ* can only be measured either simultaneously on different samples, or sequentially using a single sample, the doping level may change during the course of the combined measurements. Calculated values may then depend on the particular measurement sequence. For example, for highly doped samples with stability of just a few hours, measuring *σ* first, followed by *S* and finally *κ*, will likely overestimate *zT*, as shown in Figure 4. We recommend that claims of extraordinary performance should always be fortified by providing additional measurements conducted in an unfavorable order. Relatedly, when correlating *σ* with their corresponding *κ*, the result should be checked for consistency, since significant outliers are sometimes published without further discussion [97, 98, 99].

## 4 Conclusions

In summary, herein we argue that the field of organic thermoelectrics would benefit from the establishment of a standardized characterization protocol. We highlight prevalent issues in the literature, and propose a preliminary checklist, provided in the Supporting Information, as a starting point for addressing these challenges. We hope this perspective stimulates further discussion on the topic and contributes to the development of a robust consensus within the community.

## Funding

MCIN/AEI/10.13039/501‐100011033/ through Severo Ochoa program CEX2023‐001263‐S, Marie Skłodowska‐Curie grant agreement No 955837 – HORATES, Royal Society University Research Fellowship URF/R1/231287, Knut and Alice Wallenberg Foundation grant no. 2022.0034, National Science Foundation DMR 2310935, Marie Skłodowska‐Curie grant agreement No 101226517–FADOS, National Research Foundation of Korea grant No. RS‐2024‐00436867.

## Conflicts of Interest

The authors declare no conflicts of interest.

## Supporting information




**Supporting File**: adma72293‐sup‐0001‐SuppMat.docx.

## Data Availability

The authors have nothing to report.

## References

[1] D. Beretta , N. Neophytou , J. M. Hodges , et al., “Thermoelectrics: From History, a Window to the Future,” Materials Science and Engineering: R: Reports 138 (2019): 100501, 10.1016/j.mser.2018.09.001.

[2] “ISO 24687:2023,” accessed April, 2023, https://www.iso.org/standard/80856.html.

[3] “ISO 22007‐1:2024 and the Rest of the 22007 Subfamily of Norms,” accessed March, 2024, https://www.iso.org/standard/85974.html.

[4] S. Kommandur and S. K. Yee , “A Suspended 3‐Omega Technique to Measure the Anisotropic Thermal Conductivity of Semiconducting Polymers,” Review of Scientific Instruments 89, no. 11 (2018): 114905, 10.1063/1.5045077. 30501307

[5] U. Zhokhavets , G. Gobsch , H. Hoppe , and N. S. Sariciftci , “A Systematic Study of the Anisotropic Optical Properties of Thin Poly(3‐octylthiophene)‐Films in Dependence on Growth Parameters,” Thin Solid Films 451–452 (2004): 69–73, 10.1016/j.tsf.2003.11.042.

[6] M. Campoy‐Quiles , M. Sims , P. G. Etchegoin , and D. D. C. Bradley , “Thickness‐Dependent Thermal Transition Temperatures in Thin Conjugated Polymer Films,” Macromolecules 39, no. 22 (2006): 7673–7680, 10.1021/ma0605752.

[7] H. Zhao , Z. Li , Y. Wang , et al., “Unveiling Strong Thin Film Confinement Effects on Semirigid Conjugated Polymers,” Macromolecules 57, no. 19 (2024): 9121–9134, 10.1021/acs.macromol.4c01500. 39399832 PMC11468787

[8] V. Shrotriya , G. Li , Y. Yao , T. Moriarty , K. Emery , and Y. Yang , “Accurate Measurement and Characterization of Organic Solar Cells,” Advanced Functional Materials 16, no. 15 (2006): 2016–2023, 10.1002/adfm.200600489.

[9] E. Zimmermann , P. Ehrenreich , T. Pfadler , J. A. Dorman , J. Weickert , and L. Schmidt‐Mende , “Erroneous Efficiency Reports Harm Organic Solar Cell Research,” Nature Photonics 8, no. 9 (2014): 669–672, 10.1038/nphoton.2014.210.

[10] H. J. Snaith , “How Should You Measure Your Excitonic Solar Cells?,” Energy & Environmental Science 5, no. 4 (2012): 6513, 10.1039/c2ee03429h.

[11] “Update on the Solar Cells Reporting Summary,” Nature Energy 8, no. 12 (2023): 1299–1299, , 10.1038/s41560-023-01432-3.

[12] “Author guidelines,” Energy & Environmental Science, accessed February, 2025, https://www.rsc.org/publishing/publish‐with‐us/publish‐a‐journal‐article/energy‐and‐environmental‐science.

[13] Data Reporting Checklist, Advanced Materials, accessed February, 2025, https://advanced.onlinelibrary.wiley.com/hub/journal/15214095/author‐guidelines?tabActivePane=DataReportingChecklists.

[14] M. O. Reese , S. A. Gevorgyan , M. Jørgensen , et al., “Consensus Stability Testing Protocols for Organic Photovoltaic Materials and Devices,” Solar Energy Materials and Solar Cells 95, no. 5 (2011): 1253–1267, 10.1016/j.solmat.2011.01.036.

[15] M. V. Khenkin , E. A. Katz , A. Abate , et al., “Consensus Statement for Stability Assessment and Reporting for Perovskite Photovoltaics Based on ISOS Procedures,” Nature Energy 5, no. 1 (2020): 35–49, 10.1038/s41560-019-0529-5.

[16] I. McCulloch , A. Salleo , and M. L. Chabinyc , “Avoid the Kinks When Measuring Mobility,” Science 352, no. 6293 (2016): 1521–1522, 10.1126/science.aaf9062. 27339971

[17] H. H. Choi , K. Cho , C. D. Frisbie , H. Sirringhaus , and V. Podzorov , “Critical Assessment of Charge Mobility Extraction in FETs,” Nature Materials 17, no. 1 (2018): 2–7, 10.1038/nmat5035. 29255225

[18] H. Tang , K. Luo , and W. Chen , “Time to Standardize External Quantum Efficiency Testing of Emerging Photodetectors,” Nature Electronics 8 (2025): 874–876, 10.1038/s41928-025-01446-7.

[19] V. Pecunia , T. D. Anthopoulos , A. Armin , et al., “Guidelines for Accurate Evaluation of Photodetectors Based on Emerging Semiconductor Technologies,” Nature Photonics 19 (2025): 1178–1188, 10.1038/s41566-025-01759-1.

[20] D. Wang , J. Ding , Y. Ma , et al., “Multi‐Heterojunctioned Plastics With High Thermoelectric Figure of Merit,” Nature 632, no. 8025 (2024): 528–535, 10.1038/s41586-024-07724-2. 39048826

[21] A. Zevalkink , D. M. Smiadak , J. L. Blackburn , et al., “A Practical Field Guide to Thermoelectrics: Fundamentals, Synthesis, and Characterization,” Applied Physics Reviews 5, no. 2 (2018): 021303, 10.1063/1.5021094.

[22] K. A. Borup , J. de Boor , H. Wang , et al., “Measuring Thermoelectric Transport Properties of Materials,” Energy & Environmental Science 8 (2014): 423–435, 10.1039/C4EE01320D.

[23] R. Chetty , J. Babu , and T. Mori , “Best Practices for Evaluating the Performance of Thermoelectric Devices,” Joule 8, no. 3 (2024): 556–562, 10.1016/j.joule.2024.02.009.

[24] J. P. Heremans and J. Martin , “Thermoelectric Measurements,” Nature Materials 23, no. 1 (2024): 18–19, 10.1038/s41563-023-01726-7. 38172543

[25] J. Martin , T. M. Tritt , and C. Uher , “High Temperature Seebeck Coefficient Metrology,” Journal of Applied Physics 108, no. 12 (2010): 121101, 10.1063/1.3503505.

[26] J. Martin , “Protocols for the High Temperature Measurement of the Seebeck Coefficient in Thermoelectric Materials,” Measurement Science and Technology 24, no. 8 (2013): 085601, 10.1088/0957-0233/24/8/085601.

[27] J. Martin , W. Wong‐Ng , and M. L. Green , “Seebeck Coefficient Metrology: Do Contemporary Protocols Measure up?,” Journal of Electronic Materials 44, no. 6 (2015): 1998–2006, 10.1007/s11664-015-3640-9.

[28] J. de Boor and E. Müller , “Data Analysis for Seebeck Coefficient Measurements,” Review of Scientific Instruments 84, no. 6 (2013): 065102, 10.1063/1.4807697. 23822373

[29] Y. G. Yan , J. Martin , W. Wong‐Ng , M. Green , and X. F. Tang , “A Temperature Dependent Screening Tool for High Throughput Thermoelectric Characterization of Combinatorial Films,” Review of Scientific Instruments 84, no. 11 (2013): 115110, 10.1063/1.4830295. 24289440

[30] S. E. Yoon , B. Kim , S. Y. Chun , et al., “Impact of Molecular Weight on Molecular Doping Efficiency of Conjugated Polymers and Resulting Thermoelectric Performances,” Advanced Functional Materials 32, no. 32 (2022): 1–13, 10.1002/adfm.202202929.

[31] X. Rodríguez‐Martínez , F. Saiz , B. Dörling , et al., “On the Thermal Conductivity of Conjugated Polymers for Thermoelectrics,” Advanced Energy Materials 14 (2024): 2401705, 10.1002/aenm.202401705.

[32] F. C. Krebs , “Fabrication and Processing of Polymer Solar Cells: A Review of Printing and Coating Techniques,” Solar Energy Materials and Solar Cells 93, no. 4 (2009): 394–412, 10.1016/j.solmat.2008.10.004.

[33] H. E. Baysal , T.‐Y. Yu , V. Naenen , et al., “Omnidirectional 3D Printing of PEDOT: PSS Aerogels With Tunable Electromechanical Performance: A Playground for Unconventional Stretchable Interconnects and Thermoelectrics,” Advanced Science 12, no. 11 (2025): 1–15, 10.1002/advs.202412491. PMC11923886 39840920

[34] C. Zhang , X. Shi , Q. Liu , and Z. Chen , “Hydrogel‐Based Functional Materials for Thermoelectric Applications: Progress and Perspectives,” Advanced Functional Materials 34, no. 51 (2024): 1–27, 10.1002/adfm.202410127.

[35] A. Raouf , Y. Yu , S. Hao , et al., “Advancements in Thermoelectric Hydrogels: Structural Design and Material Innovation for Biomedical and Wearable Applications,” Journal of Materials Chemistry A 13 (2025): 41606–41652, 10.1039/D5TA05697G.

[36] L. Liu , X. Guo , D. Zhang , and R. Ma , “Thermogalvanic Hydrogels for Low‐Grade Heat Harvesting and Health Monitoring,” Materials Horizons 12, no. 15 (2025): 5473–5491, 10.1039/D4MH01931H. 40351014

[37] K. Kang , S. Watanabe , K. Broch , et al., “2D Coherent Charge Transport in Highly Ordered Conducting Polymers Doped by Solid State Diffusion,” Nature Materials 15, no. 8 (2016): 896–902, 10.1038/nmat4634. 27159015

[38] S. N. Patel , A. M. Glaudell , K. A. Peterson , et al., “Morphology Controls the Thermoelectric Power Factor of a Doped Semiconducting Polymer,” Science Advances 3, no. 6 (2017): 1700434, 10.1126/sciadv.1700434. PMC5473677 28630931

[39] K. Kang , S. Schott , D. Venkateshvaran , et al., “Investigation of the Thermoelectric Response in Conducting Polymers Doped by Solid‐State Diffusion,” Materials Today Physics 8 (2019): 112–122, 10.1016/j.mtphys.2019.02.004.

[40] L. Yu , D. Scheunemann , A. Lund , D. Kiefer , and C. Müller , “Sequential Doping of Solid Chunks of a Conjugated Polymer for Body‐Heat‐Powered Thermoelectric Modules,” Applied Physics Letters 119, no. 18 (2021): 181902, 10.1063/5.0075789.

[41] J. Liu , X. Wang , D. Li , N. E. Coates , R. A. Segalman , and D. G. Cahill , “Thermal Conductivity and Elastic Constants of PEDOT:PSS With High Electrical Conductivity,” Macromolecules 48, no. 3 (2015): 585–591, 10.1021/ma502099t.

[42] A. Weathers , Z. Ullah Khan , R. Brooke , et al., “Significant Electronic Thermal Transport in the Conducting Polymer Poly(3,4‐ethylenedioxythiophene),” Advanced Materials 27, no. 12 (2015): 2101–2106, 10.1002/adma.201404738. 25688732

[43] G.‐H. Kim , L. Shao , K. Zhang , and K. P. Pipe , “Engineered Doping of Organic Semiconductors for Enhanced Thermoelectric Efficiency,” Nature Materials 12, no. 8 (2013): 719–723, 10.1038/nmat3635. 23644522

[44] D. Simatos , I. E. Jacobs , I. Dobryden , et al., “Effects of Processing‐Induced Contamination on Organic Electronic Devices,” Small Methods 7, no. 11 (2023): 1–14, 10.1002/smtd.202300476. 37661594

[45] M. Nikolka , I. Nasrallah , B. Rose , et al., “High Operational and Environmental Stability of High‐Mobility Conjugated Polymer Field‐Effect Transistors Through the Use of Molecular Additives,” Nature Materials 16, no. 3 (2017): 356–362, 10.1038/nmat4785. 27941806

[46] G. Zuo , M. Linares , T. Upreti , and M. Kemerink , “General Rule for the Energy of Water‐Induced Traps in Organic Semiconductors,” Nature Materials 18 (2019): 588–593, 10.1038/s41563-019-0347-y. 31011215

[47] H. Wang , U. Ail , R. Gabrielsson , M. Berggren , and X. Crispin , “Ionic Seebeck Effect in Conducting Polymers,” Advanced Energy Materials 5, no. 11 (2015): 1500044, 10.1002/aenm.201500044.

[48] S. Van Reenen and M. Kemerink , “Correcting for Contact Geometry in Seebeck Coefficient Measurements of Thin Film Devices,” Organic Electronics 15, no. 10 (2014): 2250–2255, 10.1016/j.orgel.2014.06.018.

[49] G.‐H. Kim , J. Kim , and K. P. Pipe , “Humidity‐Dependent Thermoelectric Properties of Poly(3,4‐ethylenedioxythiophene):Poly(styrene sulfonate),” Applied Physics Letters 108, no. 9 (2016): 093301, 10.1063/1.4942598.

[50] R. Okazaki , A. Horikawa , Y. Yasui , and I. Terasaki , “Photo‐Seebeck Effect in ZnO,” Journal of the Physical Society of Japan 81, no. 11 (2012): 114722, 10.1143/JPSJ.81.114722.

[51] S. Yue , H. Cheng , H. He , et al., “Photo‐Enhanced Seebeck Effect of a Highly Conductive Thermoelectric Material,” Journal of Materials Chemistry A 9, no. 31 (2021): 16725–16732, 10.1039/D1TA04366H.

[52] W. Zhao , F. Zhang , X. Dai , et al., “Enhanced Thermoelectric Performance of n‐Type Organic Semiconductor via Electric Field Modulated Photo‐Thermoelectric Effect,” Advanced Materials 32, no. 31 (2020): 1–7, 10.1002/adma.202000273. 32579297

[53] Z. Ji , Z. Li , X. Dai , et al., “Photoexcitation‐Assisted Molecular Doping for High‐Performance Polymeric Thermoelectric Materials,” JACS Au 4, no. 10 (2024): 3884–3895, 10.1021/jacsau.4c00567. 39483218 PMC11522908

[54] B. Dörling , A. Hawkey , J. Zaumseil , and M. Campoy‐Quiles , “Strong Dependence of Air Stability on Thickness in n‐doped Carbon Nanotube Thermoelectrics,” Applied Physics Letters 124, no. 11 (2024): 113302, 10.1063/5.0198773.

[55] Y. Nonoguchi , M. Nakano , T. Murayama , et al., “Simple Salt‐Coordinated n‐Type Nanocarbon Materials Stable in Air,” Advanced Functional Materials 26, no. 18 (2016): 3021–3028, 10.1002/adfm.201600179.

[56] B. A. MacLeod , N. J. Stanton , I. E. Gould , et al., “Large n‐ and p‐Type Thermoelectric Power Factors From Doped Semiconducting Single‐Walled Carbon Nanotube Thin Films,” Energy & Environmental Science 10, no. 10 (2017): 2168–2179, 10.1039/C7EE01130J.

[57] C. Wang , F. Chen , K. Sun , et al., “Contributed Review: Instruments for Measuring Seebeck Coefficient of Thin Film Thermoelectric Materials: A Mini‐Review,” Review of Scientific Instruments 89, no. 10 (2018): 101501, 10.1063/1.5038406. 30399921

[58] N. Nandihalli , “A Short Account of Thermoelectric Film Characterization Techniques,” Materials Today Physics 36 (2023): 101173, 10.1016/j.mtphys.2023.101173.

[59] D. Beretta , P. Bruno , G. Lanzani , and M. Caironi , “Reliable Measurement of the Seebeck Coefficient of Organic and Inorganic Materials Between 260 and 460 K,” Review of Scientific Instruments 86, no. 7 (2015): 075104, 10.1063/1.4926885. 26233414

[60] X. He , J. Yang , Q. Jiang , et al., “A New Method for Simultaneous Measurement of Seebeck Coefficient and Resistivity,” Review of Scientific Instruments 87, no. 12 (2016): 124901, 10.1063/1.4969056. 28040937

[61] B. Dörling , O. Zapata‐Arteaga , and M. Campoy‐Quiles , “A Setup to Measure the Seebeck Coefficient and Electrical Conductivity of Anisotropic Thin‐Films on a Single Sample,” Review of Scientific Instruments 91, no. 10 (2020): 105111, 10.1063/5.0021715. 33138583

[62] V. Linseis , F. Völklein , H. Reith , K. Nielsch , and P. Woias , “Advanced Platform for the In‐Plane ZT Measurement of Thin Films,” Review of Scientific Instruments 89, no. 1 (2018): 015110, 10.1063/1.5005807. 29390699

[63] M. Craighero , Q. Li , Z. Zeng , et al., “Poly(benzodifurandione) Coated Silk Yarn for Thermoelectric Textiles,” Advanced Science 11, no. 38 (2024): 2406770, 10.1002/advs.202406770. 39099342 PMC11481370

[64] I. Miccoli , F. Edler , H. Pfnür , and C. Tegenkamp , “The 100th Anniversary of the Four‐Point Probe Technique: The Role of Probe Geometries in Isotropic and Anisotropic Systems,” Journal of Physics: Condensed Matter 27, no. 22 (2015): 223201, 10.1088/0953-8984/27/22/223201. 25985184

[65] T. Bashir , L. Fast , M. Skrifvars , and N. Persson , “Electrical Resistance Measurement Methods and Electrical Characterization of poly(3,4‐ethylenedioxythiophene)‐Coated Conductive Fibers,” Journal of Applied Polymer Science 124, no. 4 (2012): 2954–2961, 10.1002/app.35323.

[66] I. Petsagkourakis , S. Riera‐Galindo , T.‐P. Ruoko , et al., “Improved Performance of Organic Thermoelectric Generators Through Interfacial Energetics,” Advanced Science 10, no. 20 (2023): 1–12, 10.1002/advs.202206954. PMC10369274 37132565

[67] C. A. M. dos Santos , A. de Campos , M. S. da Luz , et al., “Procedure for Measuring Electrical Resistivity of Anisotropic Materials: A Revision of the Montgomery Method,” Journal of Applied Physics 110, no. 8 (2011): 083703, 10.1063/1.3652905.

[68] Q. M. Duong , D. Garcia Vidales , C. Z. Salamat , S. H. Tolbert , and B. J. Schwartz , “Measuring the Anisotropic Conductivity of Rub‐aligned Doped Semiconducting Polymer Films: The Role of Electrode Geometry,” Physical Review Applied 21, no. 2 (2024): 024006, 10.1103/PhysRevApplied.21.024006.

[69] Y. G. H‐I Un , Y. Huang , H. Sirringhaus , and I. E. Jacobs , “Unbounded Systematic Error in Thin Film Conductivity Measurements,” arXiv:2602.02418, 10.48550/arXiv.2602.02418.

[70] J.‐H. Hong , D. Kim , M.‐J. Kim , et al., “Extrapolation Method for Reliable Measurement of Seebeck Coefficient of Organic Thin Films,” Organic Electronics 108 (2022): 106582, 10.1016/j.orgel.2022.106582.

[71] H. Werheit , U. Kuhlmann , B. Herstell , and W. Winkelbauer , “Reliable Measurement of Seebeck Coefficient in Semiconductors,” Journal of Physics: Conference Series 176 (2009): 012037, 10.1088/1742-6596/176/1/012037.

[72] J. Liu , Y. Zhang , Z. Wang , et al., “Accurate Measurement of Seebeck Coefficient,” Review of Scientific Instruments 87, no. 6 (2016): 1–7, 10.1063/1.4952744. 27370476

[73] H. Y. Cai , D. F. Cui , Y. T. Li , X. Chen , L. L. Zhang , and J. H. Sun , “Apparatus for Measuring the Seebeck Coefficients of Highly Resistive Organic Semiconducting Materials,” Review of Scientific Instruments 84, no. 4 (2013): 044703, 10.1063/1.4799968. 23635216

[74] Z. Pan , Z. Zhu , J. Wilcox , J. J. Urban , F. Yang , and H. Wang , “Tackling Challenges in Seebeck Coefficient Measurement of Ultra‐High Resistance Samples With an AC Technique,” Advanced Electronic Materials 6, no. 3 (2020): 1–10, 10.1002/aelm.201901340.

[75] U. Ail , M. J. Jafari , H. Wang , T. Ederth , M. Berggren , and X. Crispin , “Thermoelectric Properties of Polymeric Mixed Conductors,” Advanced Functional Materials 26, no. 34 (2016): 6288–6296, 10.1002/adfm.201601106.

[76] H. Wang , D. Zhao , Z. U. Khan , et al., “Ionic Thermoelectric Figure of Merit for Charging of Supercapacitors,” Advanced Electronic Materials 3, no. 4 (2017): 1700013, 10.1002/aelm.201700013.

[77] H. Wang , W. Chu , and G. Chen , “A Brief Review on Measuring Methods of Thermal Conductivity of Organic and Hybrid Thermoelectric Materials,” Advanced Electronic Materials 5, no. 11 (2019): 1900167, 10.1002/aelm.201900167.

[78] D. Scheunemann and M. Kemerink , “Non‐Wiedemann‐Franz Behavior of the Thermal Conductivity of Organic Semiconductors,” Physical Review B 101, no. 7 (2020): 075206, 10.1103/PhysRevB.101.075206.

[79] R. Hanus , S. A. Gregory , M. J. Adams , S. Graham , and S. K. Yee , “Quantifying the Effects of Inhomogeneity and Doping on the Electronic Contribution to Thermal Conductivity in Semiconducting Polymers,” Advanced Electronic Materials 8, no. 11 (2022): 1–8, 10.1002/aelm.202200846.

[80] J. Guo , K. Xu , J. Asatryan , et al., “Microstructural Evolution Dominates the Changes in the Thermal Conductivity of Conjugated Polymers Upon Doping,” Advanced Functional Materials 10822 (2025): 1–11, 10.1002/adfm.202510822.

[81] W. M. Prest and D. J. Luca , “The Origin of the Optical Anisotropy of Solvent Cast Polymeric Films,” Journal of Applied Physics 50, no. 10 (1979): 6067–6071, 10.1063/1.325795.

[82] W. M. Prest and D. J. Luca , “The Alignment of Polymers During the Solvent‐Coating Process,” Journal of Applied Physics 51, no. 10 (1980): 5170–5174, 10.1063/1.327464.

[83] R. Mohan , S. Khan , R. B. Wilson , and P. E. Hopkins , “Time‐Domain Thermoreflectance,” Nature Reviews Methods Primers 5, no. 1 (2025): 55, 10.1038/s43586-025-00425-8.

[84] K. Xu , J. Guo , G. Raciti , et al., “In‐Plane Thermal Diffusivity Determination Using Beam‐Offset Frequency‐Domain Thermoreflectance With a One‐Dimensional Optical Heat Source,” International Journal of Heat and Mass Transfer 214 (2023): 124376, 10.1016/j.ijheatmasstransfer.2023.124376.

[85] X. Wang , V. Ho , R. A. Segalman , and D. G. Cahill , “Thermal Conductivity of High‐Modulus Polymer Fibers,” Macromolecules 46, no. 12 (2013): 4937–4943, 10.1021/ma400612y.

[86] R. Kroon , J. D. Ryan , D. Kiefer , et al., “Bulk Doping of Millimeter‐Thick Conjugated Polymer Foams for Plastic Thermoelectrics,” Advanced Functional Materials 27, no. 47 (2017): 1704183, 10.1002/adfm.201704183.

[87] Q. Weinbach , C. B. Nielsen , and L. Biniek , “Multi Length Scale Porosity as a Playground for Organic Thermoelectric Applications,” Journal of Materials Chemistry C 9, no. 32 (2021): 10173–10192, 10.1039/D1TC02331D.

[88] Z. Zeng , P. Sowinski , C. Müller , and B. Mihiretie , “Liquid Interface for Accurate Intrinsic Thermal Conductivity Measurements of Polymer Films Using the Transient Plane Source Method,” Journal of Thermal Science and Engineering Applications 17, no. 11 (2025): 111008, 10.1115/1.4069207.

[89] I. Brunetti , N. James Pataki , D. R. Hinojosa , et al., “A Scalable Fully Printed Organic Thermoelectric Generator for Harsh Environments Enabled by a Stable n‐Type Polymer,” Advanced Materials Technologies 10, no. 4 (2025): 1–10, 10.1002/admt.202400968.

[90] I. Brunetti , F. Ferrari , N. J. Pataki , et al., “Fully Screen‐Printed, Flexible, and Scalable Organic Monolithic Thermoelectric Generators,” Advanced Materials Technologies 9, no. 11 (2024): 2302058, 10.1002/admt.202302058.

[91] N. J. Pataki , N. Zahabi , Q. Li , et al., “A Rolled Organic Thermoelectric Generator With High Thermocouple Density,” Advanced Functional Materials 34, no. 30 (2024): 2400982, 10.1002/adfm.202400982.

[92] M. Massetti , S. Bonfadini , D. Nava , et al., “Fully Direct Written Organic Micro‐Thermoelectric Generators Embedded in a Plastic Foil,” Nano Energy 75 (2020): 104983, 10.1016/j.nanoen.2020.104983.

[93] M. Bharti , A. Singh , A. K. Debnath , et al., “Anionic Conduction Mediated Giant n‐Type Seebeck Coefficient in Doped Poly(3‐hexylthiophene) Free‐Standing Films,” Materials Today Physics 16 (2021): 100307, 10.1016/j.mtphys.2020.100307.

[94] Y. Tian , I. Florenciano , H. Xia , et al., “Facile Fabrication of Flexible and High‐Performing Thermoelectrics by Direct Laser Printing on Plastic Foil,” Advanced Materials 36, no. 15 (2024): 2307945, 10.1002/adma.202307945. 38100238

[95] F. Suarez , A. Nozariasbmarz , D. Vashaee , and M. C. Öztürk , “Designing Thermoelectric Generators for Self‐powered Wearable Electronics,” Energy & Environmental Science 9, no. 6 (2016): 2099–2113, 10.1039/C6EE00456C.

[96] A. G. Rösch , A. Gall , S. Aslan , et al., “Fully Printed Origami Thermoelectric Generators for Energy‐Harvesting,” npj Flexible Electronics 5, no. 1 (2021): 1, 10.1038/s41528-020-00098-1.

[97] I. Brunetti , A. Dash , D. Scheunemann , and M. Kemerink , “Is the Field of Organic Thermoelectrics Stuck?,” Journal of Materials Research 39 (2024): 1197–1206, 10.1557/s43578-024-01321-9.

[98] M. Wang , S. Qu , Y. Chen , Q. Yao , and L. Chen , “Monodispersed Semiconducting SWNTs Significantly Enhanced the Thermoelectric Performance of Regioregular Poly(3‐dodecylthiophene) Films,” Carbon 217 (2024): 118654, 10.1016/j.carbon.2023.118654.

[99] J. Ding , Z. Liu , W. Zhao , et al., “Selenium‐Substituted Diketopyrrolopyrrole Polymer for High‐Performance p‐Type Organic Thermoelectric Materials,” Angewandte Chemie International Edition 58, no. 52 (2019): 18994–18999, 10.1002/anie.201911058. 31605503

