# Supplementary material for: Toward a Consensus Characterization Protocol for Organic Thermoelectrics
Source: Adv Mater. 2026 Feb 5;38(14):e20430. doi: 10.1002/adma.202520430 (PMC12966978; doi:10.1002/adma.202520430)
Supplement: Supplementary file 1 — Supporting File: adma72293‐sup‐0001‐SuppMat.docx. [file ADMA-38-e20430-s001.docx]

Supporting Information

Towards a Consensus Characterization Protocol for Organic Thermoelectrics

Bernhard Dörling,^*^ Ian E. Jacobs, Irene Brunetti, Nathan James Pataki, Shannon K. Yee, Dorothea Scheunemann, Keehoon Kang, Guangzheng Zuo, Juan Sebastián Reparaz, Takao Mori, Michael L. Chabinyc, Christian Müller, Mario Caironi, Martijn Kemerink, Mariano Campoy-Quiles

## Organic thermoelectric pre-submission checklist

### General information regarding the material

- Synthesis details or supplier (incl. batch number) of dopants, organic semiconductor, carbon allotrope, etc.
- Characterization of compounds, e.g. M_w_, and M_n_ or Đ of polymers
- Sample composition including blend or composite formulation as well as any additives
- Sample processing
  - e.g. solution coating, casting, printing, melt extrusion
  - compounding method of blends or composites, where relevant
- Doping method
  - e.g. co-processing, sequential doping, ion-exchange
  - details of doping solution preparation (e.g. prepared immediately before use)
  - any post-doping washing steps (e.g. washed with X mL acetonitrile immediately after doping)
- Post-deposition treatments
  - e.g. thermal annealing, tensile drawing, rubbing
- Environment
  - processed in: (dry) air / inert atmosphere / …
  - when contamination is of particular importance, specify details up to and including e.g.:
    - glovebox levels
    - details of any solvent trap used
    - regeneration schedule
    - type/brand of pipettes used

### General information that should be supplied for all measurements

- Samples
  - Sample geometry
    - Type of substrate (including substrate dimensions) / freestanding
    - Film dimensions ± errors
    - Film roughness, porosity
    - Electrode material and deposition method
    - Electrode dimensions ± errors
    - Details on if and how the film is patterned (e.g. film outside channel is removed with toothpicks, laser ablation, photolithography, etc.)
    - Photo, microscope image, or CAD drawing of representative sample or device
      - Location of probe contact points
    - Age of sample
- Equipment / measurement method
  - Brief description of the method used
    - Name and model of commercial equipment
    - or name of the method implemented in a custom setup including reference to comprehensive description
    - e.g. for *S*: integral / differential method; transient / steady-state / quasi-steady-state / …
    - e.g. for *σ*: 4-point probe in line/ 4-bar geometry / van der Pauw / …
    - e.g. for *κ*: 3-ω / thermoreflectance / laser flash / hot-disk / …
    - e.g. for *zT*: Harman / …
  - Measurement direction
    - i.e. uniaxially in plane / randomly in plane / out-of-plane / bulk sample
  - Characteristic timescales
    - Duration of measurement / ramp rate / hold time / etc…
    - Are frequency-dependent (i.e. ionic) effects accounted for?
      - yes (specify how) / no
- Environment
  - What are the environmental conditions during measurement?
    - air (also specify humidity)/ inert atmosphere / vacuum / other …
    - in the dark / illuminated
- Statistics
  - Reproducibility
    - Number of measured devices / samples / batches
    - Have any outliers been excluded? If so, which?
  - Uncertainty
    - All independent uncertainties need to propagate and be added in quadrature
    - report value ± error, and specify what exactly each corresponds to
    - Uncertainty should be dominated by sample-to-sample variation and not the measurement uncertainty on a single sample

### Additional information specific to the Seebeck coefficient measurement

- Contacts
  - Is the contact geometry valid?
    - Physically isolated from extraneous material?
    - Size of electrical contacts is small relative to their separation?
- Temperature
  - At which temperatures is the experiment conducted?
    - specify any two of *T*_avg_, Δ*T*, *T*_hot_, *T*_cold_
  - How is the temperature measured?
    - thermocouple / evaporated resistor / IR camera / etc…
      - If IR thermometry is used, how has the emissivity of the measurement areas been determined?
  - Are temperature and voltage measured at the same spot?
    - Yes / no
      - If not, how is this accounted for?
- Thermovoltage
  - Is the voltmeter input resistance reported, and large relative to highest device resistance?
  - Is the voltmeter offset value at Δ*T* = 0 small relative to the measured thermovoltage?
    - If not, how has this offset been accounted for?
- Calibration
  - How are measurements calibrated
    - Which reference sample is used?
    - What material are the leads made of and what is their Seebeck coefficient?
- Errors
  - How is measurement error minimized? (check all that apply)
    - *V* is measured for many different values of Δ*T*
    - *V* and Δ*T* are measured at the same time on the same spot
    - *V* and Δ*T* are measured sequentially, but interpolated
    - Δ*T* is large (*V* is large)
    - Both ±Δ*T* is used to measure *V*

### Additional information specific to the electrical conductivity measurement

- Film quality
  - Is the film continuous?
  - Is the film of uniform thickness and density?
    - If not, how is *σ* calculated? Explain the model or specify assumptions made.
- Contacts
  - Is the contact geometry valid?
    - Physically isolated (e.g., cut away) from other measurement contact pads
    - *W*/*L* < 1?
    - If *W*/*L* ~ 1, are the outer contacts long?
    - If using the van der Pauw method, is the film free of holes, are contacts small, and placed in the corners?
- Correction factor
  - When calculating *σ*, are any correction factors used?
    - yes (specify) / no

### Additional information specific to the thermal conductivity measurement

- Compatibility between experiment and model
  - Is the thermal model valid for the geometry?
    - Both experiment and model consider transport to be in-plane/out-of-plane
    - If needed, both take into account anisotropy
    - Heater line width is valid for film thickness
    - Frequency range is valid for film thickness
  - Measurement mechanism (source current, probe laser, …) is not overheating the sample
    - The experimental temperature range should not exceed the modeled one to avoid unaccounted-for effects.
    - The process of measurement should not change the sample properties
- Direct vs. indirect measurement
  - When determining *κ* indirectly (i.e. via measuring the thermal diffusivity *α*), how are other required parameters determined (in this case density *ρ* and specific heat capacity *c*_p_)
    - independent measurement (specify method) / literature / …

### Additional information specific to long-term stability tests

- Repeated characterization of either single-material samples, or generators
  - Are measurements repeated to investigate stability?
    - yes / no
    - X % deviation observed after N days
  - Specify complete storage/stress protocol during repeated characterization
    - air (also specify humidity) / inert atmosphere / vacuum / …
    - temperatures
    - duration
    - other, like encapsulation, mechanical stress, …

### Information specific to the “generator” output power measurement

- Generator geometry
  - Number of connected legs/thermocouple pairs
  - What are the dimensions of the complete device?
    - length, width, thickness
  - Ratio of active vs. passive material
    - fill factor
- Heatsink
  - What type of heatsink is used?
    - No additional heatsink / external heatsink / …
  - How is ΔT maintained?
    - active cooling / natural convection / …
  - Thermal contact resistance
    - provide (an estimate for) the fraction of Δ*T* that drops over the thermoelectric material (versus the complete stack).
- Load dependence
  - Was the output measured versus a variable load?
    - measure voltage drop across a matched load resistor (i.e. maximum power point)
    - measure voltage drop across open circuit (i.e. Seebeck coefficient)
    - confirm parabolic behavior by measuring across variable load resistor
    - measure voltage at maximum power point over extended period of time to document/disprove ionic contributions
- Normalized performance
  - What are the power densities (power / area) and TEG power factors (power / area / temperature²)?
    - Calculate densities using the total (i.e. not just the active) device area
    - When reporting the TEG power factor, specify explicitly if the full applied Δ*T*_full_ was used for the calculation, or only the fraction Δ*T*_active_, dropping across the active material.

### Consistency checks

- Comparability across samples and methods
  - Is evidence provided that different types of samples can reasonably be assumed to have similar properties?
    - yes / no
  - Is potential anisotropy accounted for?
    - yes / no
    - Are different properties measured in the same direction?
- Has the possibility of sample evolution during or between measurements (dedoping, degradation, exposure to harsh conditions during measurement, etc.) been characterized?
  - yes (specify how) / no
  - When multiple characterization techniques are applied to a single sample, specify the order of measurements
  - Are all measurements done on samples of the same “freshness” (e.g. with regards to doping)?
    - yes / no
    - If not, how is this accounted for when correlating *S*, *σ*, *κ*, or when calculating *PF* or *zT*?
- Bare materials vs. generator
  - Is the provided data sufficient to compare the characteristics of the constituent p- and n-type active materials to the performance of the generator?
    - yes / no
    - Comparison shows X % deviation
  - Estimate generator contact resistance
    - Compare total generator resistance to expected resistance of all individual legs
